# HLA Class II Defects in Burkitt Lymphoma: Bryostatin-1-Induced 17 kDa Protein Restores CD4+ T-Cell Recognition

**DOI:** 10.1155/2011/780839

**Published:** 2011-11-28

**Authors:** Azim Hossain, Jason M. God, Faisal F. Y. Radwan, Shereen Amria, Dan Zhao, Jennifer R. Bethard, Azizul Haque

**Affiliations:** ^1^Department of Microbiology and Immunology, Medical University of South Carolina, 173 Ashley Avenue, Charleston, SC 29425, USA; ^2^Hollings Cancer Center, Medical University of South Carolina, 173 Ashley Avenue, Charleston, SC 29425, USA; ^3^Charles P. Darby Children's Research Institute, Medical University of South Carolina, 173 Ashley Avenue, Charleston, SC 29425, USA; ^4^Department of Cell and Molecular Pharmacology and Experimental Therapeutics, Medical University of South Carolina, 173 Ashley Avenue, Charleston, SC 29425, USA

## Abstract

While the defects in HLA class I-mediated Ag presentation by Burkitt lymphoma (BL) have been well documented, CD4+ T-cells are also poorly stimulated by HLA class II Ag presentation, and the reasons underlying this defect(s) have not yet been fully resolved. Here, we show that BL cells are deficient in their ability to optimally stimulate CD4+ T cells via the HLA class II pathway. The observed defect was not associated with low levels of BL-expressed costimulatory molecules, as addition of external co-stimulation failed to result in BL-mediated CD4+ T-cell activation. We further demonstrate that BL cells express the components of the class II pathway, and the defect was not caused by faulty Ag/class II interaction, because antigenic peptides bound with measurable affinity to BL-associated class II molecules. Treatment of BL with broystatin-1, a potent modulator of protein kinase C, led to significant improvement of functional class II Ag presentation in BL. The restoration of immune recognition appeared to be linked with an increased expression of a 17 kDa peptidylprolyl-like protein. These results demonstrate the presence of a specific defect in HLA class II-mediated Ag presentation in BL and reveal that treatment with bryostatin-1 could lead to enhanced immunogenicity.

## 1. Introduction

Burkitt lymphoma (BL) is an aggressive non-Hodgkin's B-cell malignancy, occurring most frequently as endemic BL in children living in areas of high malarial prevalence [1]. This malignancy may also be found in other parts of the world as sporadic BL and accounts for 1-2% of all lymphomas in Western countries [1]. The clinical manifestations of BL are variable, with tumors of the jaw characteristically seen in endemic BL and tumors in the gut associated with sporadic BL [2–4]. BL has one of the fastest doubling times among human malignancies and is frequently associated with immune deficiency [3]. 

In addition to its strong association with malaria, BL has a high correlation with Epstein-Barr Virus (EBV). EBV infection, however, is not requisite for the development of BL, and the degree of association with EBV varies based on the type of BL. EBV infection occurs in >90% of endemic BL cases, 10–15% of sporadic BL, and 40% of human immunodeficiency virus (HIV) associated BL [1]. While the exact role that EBV plays in the development of BL remains largely unknown, it is understood that EBV gene products may be involved in the transformation of BL cells and their decreased immunogenicity. Additional evidence for EBV having a role in development of BL stems from EBV's link to various other lymphoid malignancies including Hodgkin's lymphoma, transplant-related B-cell lymphomas, T-cell lymphomas, adult T-cell leukemia, and natural killer cell leukemia [5–8]. While BL has varying associations with malaria and EBV and in some cases is not associated with either, the one feature shared by all BLs is overexpression of the oncogenic transcription factor *c-myc*, which has a gene network comprising up to 15% of all known genes [9]. This abnormality results from the translocation of the *MYC* gene to an immunoglobulin locus leading to its constitutive activation [10–12].

BL is known to be deficient in HLA class I-mediated antigen (Ag) presentation to CD8+ T lymphocytes [13–15]. However, the role of HLA class II-mediated Ag presentation in generating an immune response to BL has not been fully elucidated. The class I defect has been well studied and is understood to result from the weak immunogenicity of EBV nuclear Ag 1 (EBNA1), which is poorly processed and presented through the class I pathway [16–18]. Another EBV gene product, gp42, has a role in mediating virus binding through interaction with HLA class II and it has been speculated to block the interaction between class II and the T-cell receptor [19, 20]. Although HLA class I-mediated activation of CD8+ T cells leads to Ag-specific lysis of tumor cells, an HLA class II response is vital for the generation of sustained immune responses [21]. Our laboratory has previously shown that B-cell lymphomas are deficient in HLA class II-mediated Ag presentation [22], and in this study we explore the role of B-cell-associated molecules in restoration of CD4+ T-cell recognition of BL cells.

The study presented here suggests that multiple defects may contribute to BL's inability to efficiently present Ag via HLA class II molecules. We confirm expression of a transfected HLA class II allele in both BL cells and EBV-immortalized B-lymphoblastoid cells (B-LCL), and demonstrate that the transfected HLA class II efficiently binds exogenously delivered Ag to form class II peptide complexes. However, while B-LCL were capable of CD4+ T-cell stimulation, BL cells were deficient in their ability to do so, and addition of external co-stimulation was insufficient to overcome this defect. In addition, treatment of BL cells with bryostatin-1 partially restored class II-mediated Ag presentation. This restoration was linked to the upregulation of a 17 kDa protein in bryostatin-treated BL which was expressed at low levels in untreated BL but highly expressed in B-LCL, suggesting that this protein may play a role in enhancing class II-mediated Ag presentation. In other studies, bryostatin-1 has been shown to increase HLA class II expression in dendritic cells and in a colorectal carcinoma cell line, but its effect on HLA class II expression and Ag presentation in lymphoid malignancies has not previously been evaluated [23, 24]. On the whole, these results suggest that BL possesses multiple defects which lead to an impaired ability to stimulate CD4+ T cells through HLA class II Ag presentation. These defects may provide the opportunity to develop novel immunotherapies leading to more targeted treatment of BL and other lymphoid malignancies. This study also provides a rationale for the further evaluation of bryostatin-1 as a therapeutic treatment of lymphoid malignancies.

## 2. Materials and Methods

### 2.1. Cell Lines

Human BL cell lines, Nalm-6, Ramos, and Ous, were maintained in complete RPMI-1640 supplemented with 10% fetal bovine serum (Invitrogen, Carlsbad, CA), 50 U/mL penicillin 50 *μ*g/mL streptomycin, and 1% L-glutamine (Mediatech, Manassas, VA). The Ous cell line was a gift from Dr. Christian Munz (Rockefeller University). The human B-lymphoblastoid cell lines (B-LCL) 6.16 and Frev were maintained in IMDM supplemented with 10% bovine growth serum (Hyclone, Logan, UT), 50 U/mL penicillin 50 *μ*g/mL streptomycin, and 1% L-glutamine (Mediatech). Nalm-6, Ramos, and 6.16 cells were retrovirally transfected for constitutive expression of HLA-DR4 (DRB1*0401) with linked drug selection markers for hygromycin and histidinol resistance to generate Nalm-6.DR4, Ramos.DR4, and 6.16.DR4 [22, 25]. Frev did not require transfection of the class II allele as it constitutively expresses HLA-DR4. Surface HLA-DR4 expression in the transfectants was confirmed by flow cytometric analysis using the DR4-specific mAb, 359-F10 [22, 26, 27]. 6.16.DR4 cells were further transfected with DM*α* and DM*β* for constitutive expression of HLA-DM molecules to generate 6.16.DR4.DM [22]. The expression of HLA-DM on 6.16.DR4.DM cells was confirmed by western blotting. T-cell hybridomas 2.18a and 1.21 recognize Ig *κ* residues 188–203 and 145–159, respectively, and were generated by immunization of DR4-transgenic mice as described [25, 28]. The T-cell hybridoma 17.9 (generously provided by D. Zaller, Merck Research Laboratories, Rahway, NJ) responds to human serum albumin (HSA) residue 64–76 K [29]. These T cell hybridomas are less dependent on costimulatory signals for their stimulation. Cells were cultured in RPMI 1640 with 10% FBS, 50 U/mL penicillin, 50 *μ*g/mL streptomycin, and 50 *μ*M *β*-mercaptoethanol (Invitrogen).

### 2.2. Antigens, Peptides, and Other Reagents

Human serum albumin (HSA) and human IgG *kappa* (IgG *κ*) were purchased from Sigma (St. Louis, MO). HSA_64–76 K_ peptide (sequence: VKLVNEVTEFAKTK) human IgG immunodominant peptide *κ*_188–203_ (*κ*I; sequence: KHKVYACEVTHQGLSS), and subdominant peptide *κ*_145–159_ (*κ*II; sequence: KVQWKVDNALQSGNS) were produced using Fmoc technology and an Applied Biosystems Synthesizer as described, dissolved in PBS, and stored at −20°C until used [25, 29, 30]. Reverse phase HPLC purification and mass spectrometry were used to analyze the peptide and showed a peptide purity >99%. Bryostatin-1 was purchased from Sigma.

### 2.3. Antigen Presentation Assays

B-LCL and BL were incubated with 0 *μ*M, 5 *μ*M, 10 *μ*M, or 20 *μ*M HSA Ag or HSA synthetic peptide for 3–24 h at 37°C in the appropriate cell culture media to determine optimal antigen concentrations to use in antigen presentation assays [22, 25]. Following titration, the same assays were carried out using only the optimal concentration of each antigen. Cells were then washed and co-cultured with the T-cell hybridoma 17.9 for 24 h at 37°C. In parallel assays, 2.18a and 1.21 were stimulated with anti-CD3/CD28 prior to co-culture with Nalm-6.DR4, Ramos.DR4, or 6.16.DR4.DM which had been incubated with *κ*I or *κ*II [29]. Following co-culture, T cell production of IL-2 was quantitated by ELISA [31]. Assays were repeated in triplicate with standard error for triplicate samples within a single experiment being reported.

### 2.4. Western Blotting

Western blot analysis was performed on whole cell lysates of Frev, Nalm-6.DR4, 6.16.DR4.DM, and Ramos.DR4. Expression of HLA class II, Ii, and HLA-DM was analyzed as described previously [32, 33]. Densitometry was performed using a ChemiDoc XRS station (Bio-Rad) where the protein bands were analyzed using the Quantity One 4.6.3 software (Bio-Rad). Relative protein expression levels were stated as a ratio of specific proteins expressed/*β*-actin for each sample. Data are representative of at least three separate experiments.

### 2.5. IL-2 ELISA

IL-2 levels in Ag presentation assay supernatant were quantitated by ELISA. A 96-well ELISA plate was coated overnight at 4°C with purified rat anti-mouse IL-2 (Sigma). The plate was then washed and blocked with 2% BSA at RT for 30 m. After washing, standards and samples were plated in appropriate wells and incubated at RT for 2 h. A standard curve was generated using recombinant IL-2 purchased from R&D (Minneapolis, MN). The plate was washed, and biotinylated rat anti-mouse IL-2 (Sigma) was added and incubated at RT for 1 h. Following washing, avidin peroxidase (Pierce, Rockford, IL) was added to each well and incubated at RT for 30 m. The plate was washed, and PNPP substrate (Thermo Scientific, Rockford, IL) was added to each well and incubated at RT. Readings were taken every 30 m at 405 nm. IL-2 levels in sample wells are expressed in pg/mL, calculated from the standard curve. Assays were repeated in triplicate and expressed as mean IL-2 ± SEM.

### 2.6. Peptide Binding Assays

Nalm-6.DR4, Ramos.DR4, 6.16.DR4.DM, and Frev cells were fixed in 1% paraformaldehyde and then incubated overnight with 0 *μ*M, 10 *μ*M, or 20 *μ*M biotinylated HSA peptide (b-HSA) in 150 mM CPB (pH 7.4), washed with PBS, and lysed on ice for 20 min with 50 mM Tris buffer (pH 8) containing 0.15 M NaCl and 0.5% IGEPAL CA 630 (Sigma) as described [30, 34]. Cell supernatants were added to plates (Costar, Cambridge, MA) previously coated overnight with the anti-human class II antibody 37.1 (kindly provided by L. Wicker, Merck Research Lab, Rahway, NJ). The captured class II-peptide complexes were detected with europium-labeled streptavidin (Pharmacia Fine Chemicals, Piscataway, NJ) using a fluorescence plate reader (Delfia, Wallac, Turku, Finland). The number of total DR molecules within B-LCL/BL cells was quantitated as described [28].

### 2.7. Bryostatin-1 Treatment of BL Cells

Nalm-6.DR4 and Ramos.DR4 were treated with 0, 20, or 40 nM of bryostatin-1 for overnight. Following incubation, untreated and bryostatin-treated cells were used in Ag presentation assays with HSA peptide followed by ELISA IL-2 quantitation as already described. In separate assays, Ramos.DR4 were treated with 40 nM bryostatin overnight and then fixed in 1% paraformaldehyde for 5 min. Following fixation, the cells were washed and incubated with 0 *μ*M, 10 *μ*M, or 40 *μ*M of b-HSA for 3 h at 37°C with shaking. The cells were then washed and lysed in Hanks Balanced Salt Solution with 1% Triton-X-100 and protease inhibitors (PMSF and TLCK). The lysate was added to wells of a 96-well plate precoated with the anti-HLA class II antibody, 37.1. Captured class II peptide complexes were detected using streptavidin peroxidase and BD OptEIA TMB substrate reagents (BD, San Diego, CA). The reaction was stopped using 1 M phosphoric acid and the resulting absorbance was read at 450 nM.

Additionally, surface protein expression in 6.16.DR4.DM, untreated Nalm-6.DR4, and bryostatin-treated Nalm-6.DR4 was evaluated by SDS-PAGE protein separation. Nalm-6.DR4 cells were treated overnight with 40 nM of bryostatin-1. Following incubation, 6.16.DR4.DM, untreated Nalm-6.DR4, and bryostatin-treated Nalm-6.DR4 were washed in citrate phosphate buffer (CPB) to elute cell surface proteins. The resulting eluate was then subjected to SDS-PAGE. A 17 kDa band was excised from these gels and analyzed by MALDI TOF-TOF mass spectrometry. Proteins in CPB eluate from 6.16.DR4.DM were separated by electrophoresis on large, nonreducing gels. The 17 kDa band was excised and the protein was extracted by sonication in PBS on ice. Ramos.DR4 cells were incubated with the HSA peptide in the 17 kDa extract for use in Ag presentation assays as described. T cell production of IL-2 was quantitated [31].

### 2.8. Protein Extraction and Digestion

CPB eluate was obtained from 6.16.DR4.DM, untreated Nalm-6.DR4, and bryostatin-treated Nalm-6.DR4 as described previously [22]. Extracts were concentrated, and protein concentrations were measured, then run on a non-reducing gel, and stained with Coomassie blue. Gel plugs were excised and placed in an eppendorf tube. Each plug was washed with 50 mM ammonium bicarbonate for 10 minutes. Next, the plugs were destained using 25 mM ammonium bicarbonate in 50% acetonitrile for 15 minutes. The plugs were dehydrated with 100% acetonitrile for 15 minutes and dried in a speedvac. Each gel plug was covered with Proteomics Grade Trypsin (Sigma) and incubated at 37°C overnight. The supernatant was collected in a clean dry eppendorf tube. Peptides were further extracted with 1 wash of 25 mM ammonium bicarbonate for 20 minutes and three washes of 5% formic acid, 50% acetonitrile for 20 minutes each. The supernatent was collected and pooled after each wash then dried down in a speedvac to ~1 uL. Prior to analysis, the samples were reconstituted with 10 uL of 0.1% trifluoroacetic acid. Samples were then concentrated with a C18 Ziptip (Millipore) and eluted with 0.1% TFA, 50% acetonitrile, and 7.0 mg/mL *α*-cyano-4-hydroxycinnamic acid directly onto the MALDI target.

### 2.9. Mass Spectrometric Analysis (MALDI TOF/TOF)

After the spots were dried completely, the plate was loaded into the Applied Biosystems 4800 Proteomics Analyzer. An external calibration was performed prior to analyzing samples utilizing the manufacturer's standards and protocols. Samples were analyzed in batch mode using 2000 laser shots per spectrum. First, peptide mass maps were acquired over the m/z range of 800–3500 in reflectron mode with a delayed extraction time optimized for m/z 2000 by averaging 2000 scans to locate peaks of peptide origin. The next batch run performed MS-MS analyses to obtain sequence data on the 20 most abundant peaks from the MS analysis. Upon completion of the batch processing, the data was exported into the GPS Explorer data processing system for interpretation and identification. The MASCOT database-searching algorithm analyzed the data and summarized the results in report format. Database searches were performed using 2 missed cleavages and one differential modification of methionine oxidation. The top 20 matches were reviewed prior to assigning confident protein identifications.

## 3. Results

### 3.1. BL Cells Display Decreased HLA Class II-Mediated CD4+ T-Cell Stimulation

**Figure 1:**
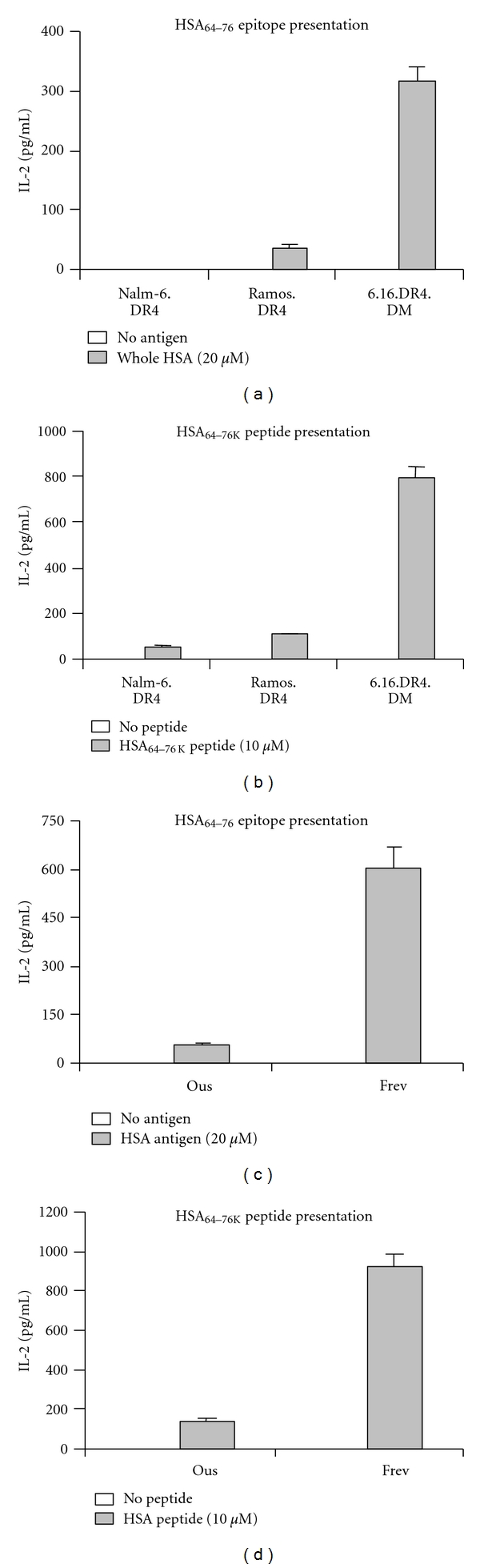
BLs are deficient in their ability to present Ag in the context of HLA class II. BL (Nalm-6.DR4, Ramos.DR4, and OUS) and B-LCL (6.16.DR4.DM and Frev) were incubated with whole HSA (a and c) or HSA synthetic peptide (b and d). Following incubation, cells were washed and co-cultured with the HSA_64–76 k_ epitope-specific T-cell hybridoma 17.9. Supernatant from the co-culture was assayed by ELISA to determine IL-2 levels as a measure of T-cell stimulation. All three BL cell lines were deficient in stimulation of IL-2 production for both whole HSA and HSA synthetic peptide, while both B-LCL efficiently presented each Ag to stimulate high levels of IL-2 production.

Although BL and B-LCL both express surface HLA class II, we transfected these cell lines to express a common class II allele so that we might obtain a more direct comparison of class II-mediated Ag presentation between the two cell types. Retroviral gene transfections of a DR4 allele, HLA DRB1*0401, were carried out on our BL (Nalm-6 and Ramos) and B-LCL (6.16) cell lines. Flow cytometric analysis confirmed transfection and expression of this allele in all three cell lines (data not shown). 6.16.DR4 cells were additionally transfected with HLA-DM to generate 6.16.DR4.DM cells expressing similar levels of HLA-DM when compared to Nalm-6.DR4 and Ramos.DR4 [33]. Transfectants were then sorted, matched for surface DR4 expression, and incubated, along with Ous and Frev, in culture media with either HSA antigen or HSA peptide. Following incubation, cells were washed and co-cultured with the T-cell hybridoma, 17.9, for 24 h at 37°C. Culture supernatant was collected and assayed by ELISA for IL-2 levels. The results of these assays demonstrate that Nalm-6.DR4, Ramos.DR4, and Ous were deficient in their ability to stimulate IL-2 production in 17.9 by class II-mediated presentation of HSA epitope or HSA synthetic peptide (Figures 1(a)–1(d)). The B-LCL lines 6.16.DR4.DM and Frev, however, stimulated production of high levels of IL-2 (Figures 1(a)–1(d)). Supplemental Figure  1 (see Supplemental material available online at doi:10.1155/2011/585893) shows the results of whole HSA and HSA peptide titration with Nalm-6.DR4, Ramos.DR4 and 6.16.DR4.DM. Nalm-6.DR4 and Ramos.DR4 fail to stimulate IL-2 production at all concentrations of whole HSA or HSA peptide, while 6.16.DR4.DM shows a dose-dependent increase in levels of IL-2 production. These results suggest that BL cells possess a defect(s) in the presentation of Ag to CD4+ T cells in the context of HLA class II.

### 3.2. BL and B-LCL Express Similar Levels of HLA Class II Pathway Components

**Figure 2:**
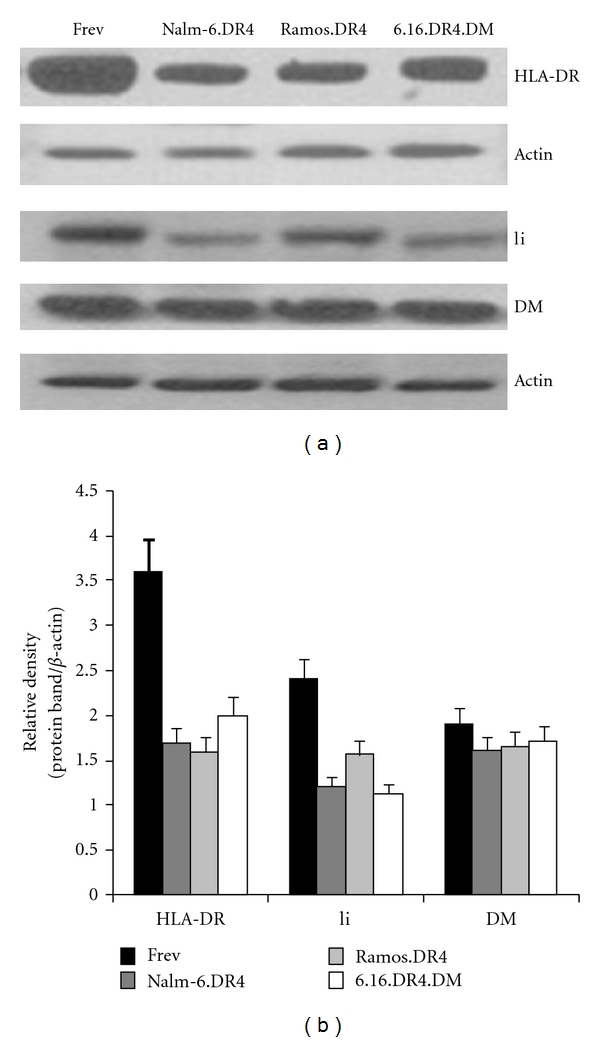
BL and B-LCL express similar levels of HLA class II pathway components. Variations in efficiency of Ag presentation between BL and B-LCL may be attributable to differences in expression levels of components in the class II pathway. To evaluate this possibility, the BL cell lines Nalm-6.DR4 and Ramos.DR4, the B-LCL cell lines 6.16.DR4.DM and Frev were analyzed by western blotting for expression of HLA class II, invariant chain (Ii), and HLA-DM (a). Densitometric analysis confirmed the expression of comparable levels of each of these class II pathway components (b).

Western blot analysis was performed on Nalm-6.DR4, Ramos.DR4, 6.16.DR4.DM, and Frev for expression of HLA class II, Ii, and HLA-DM. Data from these analyses revealed that both BL and B-LCL expressed detectable levels of these immune components (Figure 2(a)). As a wild type B-LCL, Frev expresses higher levels of class II pathway components than Nalm-6.DR4, Ramos.DR4, and 6.16.DR4.DM, as analyzed by densitometry and corrected for actin loading controls (Figure 2(b)). These data suggest that the observed defect in class II-mediated Ag presentation by BL is not the result of a defect in the HLA class II processing and presentation pathway.

### 3.3. Addition of External Co-Stimulation Is Insufficient to Overcome the BL-Associated Defect in Class II-Mediated Ag Presentation

**Figure 3:**
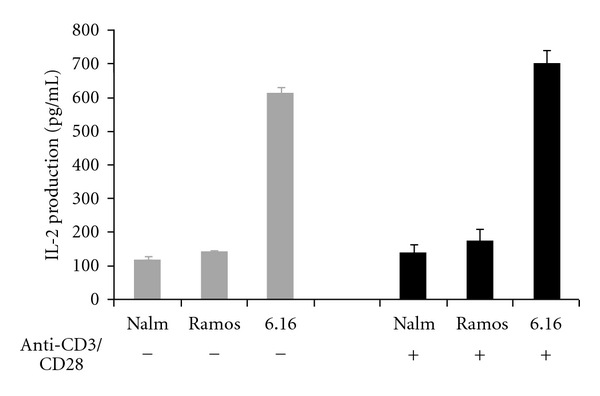
Addition of external co-stimulation is not sufficient to overcome class II-associated defects in BL. BLs are known to express lower levels of costimulatory molecules, raising the possibility that this was the cause of the observed defect in their ability to present Ag via class II. BL cell lines Nalm-6.DR4 and Ramos.DR4, and the B-LCL cell line 6.16.DR4.DM were incubated with *κ*I and *κ*II peptides prior to co-culture with the T-cell hybridomas 2.18a or 1.21 which had been stimulated with anti-CD3/CD28. Culture supernatant was assayed by ELISA for IL-2 levels as a measure of T-cell stimulation. These results demonstrate that Nalm-6.DR4 and Ramos.DR4 Ag presentation is unaffected by the addition of external co-stimulation and remains deficient in class II-mediated presentation.

It has previously been reported that BL cells are deficient in expression of co-stimulatory molecules (CD80/86). In order to determine if this was the cause of the defect in class II-mediated Ag presentation by BL, Ag presentation assays were performed in the presence of external co-stimulatory signals as described. In this assay, T-cell hybridomas were treated with anti-CD3/CD28 plus cross-linked IgG and co-cultured with the BL cells that were preincubated with HSA peptide. The addition of external co-stimulation had little to no effect on class II Ag presentation by BL (Figure 3). While 6.16.DR4.DM stimulated high levels of IL-2 production in T cells with or without external co-stimulation, Nalm-6.DR4 and Ramos.DR4 showed no significant increase in stimulation of IL-2 production in T cells with external co-stimulation.

### 3.4. HSA Peptide Binds with Similar Affinity to HLA Class II on BL and B-LCL

**Figure 4:**
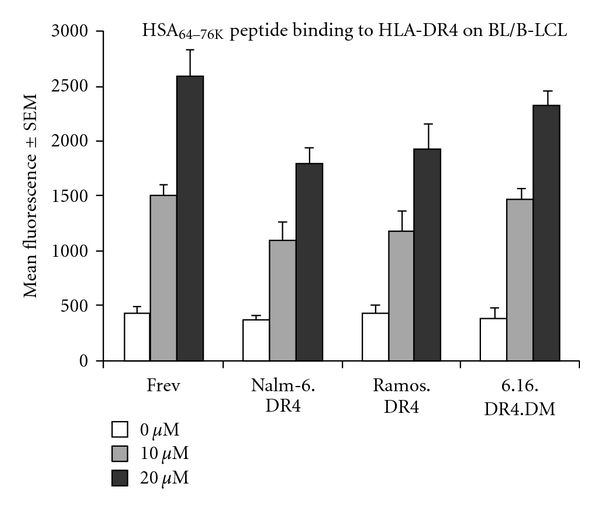
BL and B-LCL bind HSA_64–76 K_ synthetic peptide with similar affinity. Ag presentation depends on efficient binding of Ag to HLA class II proteins. BL cell lines Nalm-6.DR4 and Ramos.DR4, and the B-LCL lines 6.16.DR4.DM and Frev were fixed and incubated with biotin-labeled HSA_64–76 K_ at pH 7.4. Cells were lysed and class II peptide complexes were detected in ELISA format with europium-labeled streptavidin with mean fluorescence used as a measure of peptide binding. Each cell line bound similar levels of peptide.

The next step in evaluating the BL-related defect in HLA class II Ag presentation was to assess the binding efficiency of HSA peptide to BL-expressed surface DR4. Nalm-6.DR4, Ramos.DR4, 6.16.DR4.DM, and Frev were incubated with various concentrations of b-HSA at pH 7.4. Class II peptide complexes were then detected in an ELISA format using europium-labeled streptavidin. Data showed a dose-dependent response with each cell line binding b-HSA peptide with a similar, measurable affinity (Figure 4). These results suggest that BL's reduced capacity to present Ag via HLA class II is not a result of impaired peptide binding to HLA class II molecules.

### 3.5. Bryostatin Treatment of BL Increases Peptide Binding to HLA Class II and Restores Class II Ag Presentation and CD4+ T-Cell Recognition

**Figure 5:**
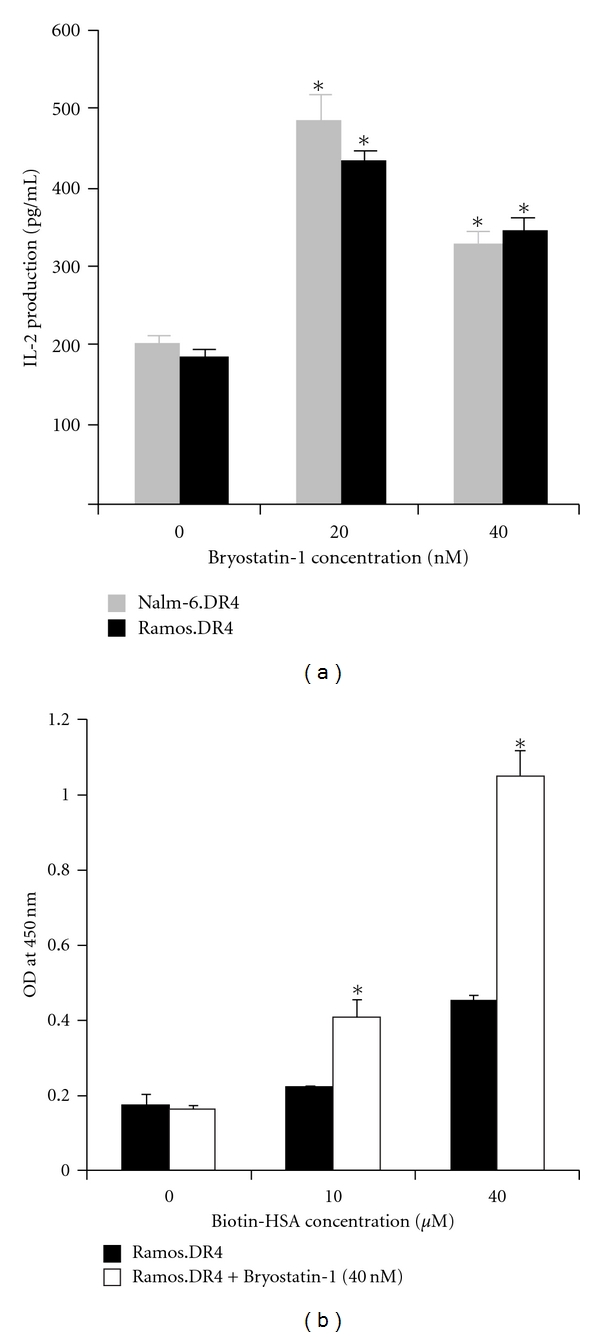
Bryostatin treatment increased class II peptide binding and restored class II Ag presentation in BL cell lines. (a) The BL cell lines Nalm-6.DR4 and Ramos.DR4 were treated with 0, 20, or 40 nM bryostatin-1 for 24 h. Following treatment, cells were collected, washed, and incubated with HSA_64–76 K_ synthetic peptide for 24 h. Cells were then washed and co-cultured with the T-cell hybridoma 17.9. Supernatant from the co-culture was assayed by ELISA to determine IL-2 levels as a measure of T-cell stimulation. Results from these assays show that bryostatin-1 treatment significantly restores Ag presentation in both Nalm-6.DR4 and Ramos.DR4 to levels comparable to B-LCL. (b) Ramos.DR4 cells were treated with 40 nM bryostatin overnight and then washed, fixed, and incubated with 0 *μ*M, 10 *μ*M, or 40 *μ*M of b-HSA for 3 h at 37°C with shaking. The cells were then collected and lysed, and class II/peptide complexes were detected by ELISA. *P < 0.001.

Previous studies on bryostatin-1 have shown that it causes upregulation of HLA class II molecules in the professional Ag presenting dendritic cells and leads to increased T-cell stimulation by these cells [23]. Based on this finding, we sought to determine if bryostatin-1 treatment would impact Ag presentation by BL. Nalm-6.DR4 and Ramos.DR4 cells were treated with bryostatin-1 overnight and then used in Ag presentation assays with HSA as already described. Untreated BL cells showed similarly low levels of T-cell stimulation, whereas cells treated with bryostatin-1 at 20–40 nM restored Ag presentation and T-cell stimulation (Figure 5(a)). Ramos.DR4 cells were treated with bryostatin-1 overnight, and peptide binding to HLA class II was measured as already described. Ramos.DR4 treated with 40 nM bryostatin-1 showed significantly higher levels of peptide binding at both 10 *μ*M and 40 *μ*M b-HSA (Figure 5(b)).

### 3.6. Bryostatin Treatment Upregulates Expression of an Immunostimulatory 17 kDa Protein in BL

**Figure 6:**
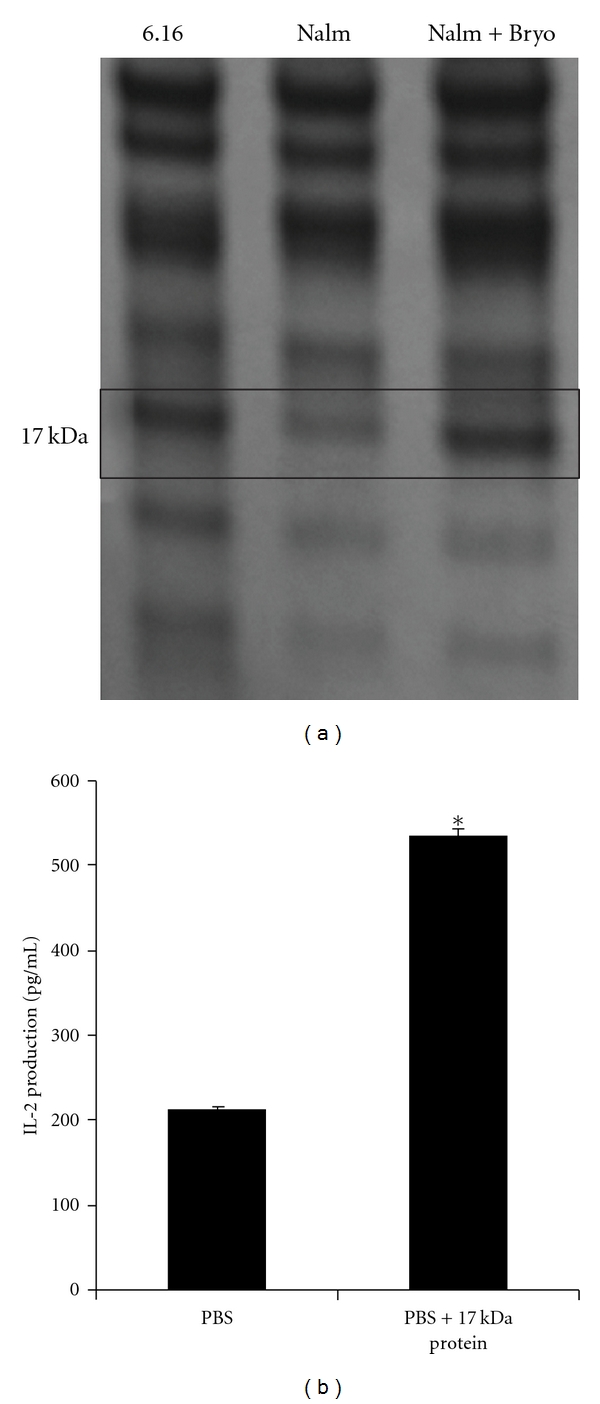
Treatment of BL with bryostatin-1 causes upregulation of a 17 kDa immunostimulatory protein. (a) Acid eluate from Nalm-6.DR4, Nalm-6.DR4 treated for 24 h with 40 nM bryostatin-1 and 6.16.DR4.DM were collected, and subjected to SDS-PAGE, followed by coomassie blue staining. Gel banding patterns revealed upregulation of a 17 kDa protein in bryostatin-treated Nalm-6.DR4 that is expressed at low levels in untreated Nalm-6.DR4 but highly expressed in 6.16.DR4.DM. (b) Acid eluate from 6.16.DR4.DM was separated on a large, non-reducing gel, the band corresponding to 17 kDa was excised, and the protein was extracted by sonication in PBS on ice. Ramos.DR4 cells were then incubated in PBS or 17 kDa gel extract with HSA_64–76 K_ synthetic peptide. Cells were then washed and co-cultured with the T-cell hybridoma line 17.9. Supernatant from the co-culture was assayed by ELISA to determine IL-2 levels as a measure of T-cell stimulation. Results show that IL-2 stimulation is significantly increased in Ramos.DR4 cells incubated with peptide in the presence of the 17 kDa gel extract. *indicates *P* < 0.05.

To determine the nature of the class II presentation restoration in BL following bryostatin-1 treatment, protein expression patterns in CPB eluates from Nalm-6.DR4 and 6.16.DR4.DM were analyzed by gel electrophoresis (non-reducing gel) and coomassie blue staining. This study showed that a 17 kDa protein was consistently expressed at low levels in BL cells (Nalm-6.DR4) but high levels in B-LCL (6.16.DR4.DM) cells (Figure 6(a)). Following overnight treatment of BL cells with bryostatin-1, expression of this 17 kDa protein was restored to levels comparable to 6.16.DR4.DM cells (Figure 6(a)). This protein band was then cut from the gel and analyzed by MALDI TOF/TOF mass spectrometry, revealing a peptidylprolyl-like protein (accession number: 89058151). To further analyze the function of this 17 kDa protein, CPB eluates from 6.16.DR4.DM cells or bryostatin-1-treated BL cells were separated on a large non-reducing gel, the band corresponding to 17 kDa protein was excised, and the protein was extracted by sonication in PBS on ice. Ramos.DR4 cells were then incubated with HSA peptide (10 *μ*M) in the presence of this extract, followed by washing and co-culture with 17.9 T cells. ELISA IL-2 quantitation of the assay supernatant showed a significant increase in the stimulation of IL-2 production by Ramos.DR4 cells incubated with HSA in the presence of the 17 kDa extract (Figure 6(b)). These results suggest that bryostatin-1 treatment upregulates expression of a 17 kDa protein in BL, and this protein has an immunostimulatory function.

## 4. Discussion

BL possesses a well-known defect in HLA class I-mediated Ag presentation, resulting from the poor immunogenicity of the EBNA1 protein. EBNA1 possesses an internal Gly-Ala repeat that impairs its proteasomal processing, leading to weak stimulation of CD8+ T cells [35]. This defect, although well studied, addresses only one aspect of the immune response to BL. Less is known about the role of HLA class II-mediated immune responses to this malignancy. Studies have generally focused on CD8+ T-cell responses due to their ability to directly kill target cells, but CD4+ T-cell responses mediated by class II are needed for lasting immune responses and memory [36, 37].

In this study, we have shown that although BLs express measurable class II proteins on their cell surface, they were unable to stimulate CD4+ T cells through presentation of HSA peptide or epitope. We demonstrate further that when incubated in buffer at pH 5.5, BL cells regain class II-mediated Ag presentation capacity. Treatment of BL with bryostatin-1 led to restoration of class II presentation and CD4+ T-cell stimulation. This restoration was due, in part, to the upregulation of a 17 kDa, immunostimulatory, peptidylprolyl-like protein which is normally expressed at very low levels in BL, but highly expressed in B-LCL.

The efficiency of class II-mediated Ag presentation to CD4+ T cells may be partially affected by the expression levels of components in the class II pathway: Ii (invariant chain), HLA-DM and HLA-DO [26, 33, 38]. However, we did not observe any significant differences in the expression levels of these pathway components between two BL and two B-LCL cell lines, ruling this out as contributing to the observed defect in BL. CD4+ T cell activation is also dependent on signals delivered by the co-stimulatory molecules, CD80/86, expressed by B cells, yet BLs are known to express lower levels of these molecules [39]. It is plausible that the BL-associated class II defect was a result of insufficient co-stimulation. External co-stimulation may be provided to T cells in the form of anti-CD28, which serves as a surrogate for CD80/86. While our T-cell hybridomas do not require co-stimulation, we still evaluated whether the decreased expression of co-stimulatory molecules contributed to the BL defect. However, even under these conditions, BL cells were unable to stimulate activation of CD4+ T cells. We gleaned further evidence that co-stimulation is not the cause the BL-associated class II defect from assays demonstrating that cross-linking IgM on BL cells failed to result in CD4+ T-cell stimulation (data not shown).

The presentation of Ag via HLA is central to the immune system's ability to detect pathogens and transformed cells and mount immune responses to these cells. Efficient Ag presentation is dependent on efficient binding of peptides to HLA. While co-stimulation was not the cause of BL's inability to present Ag through class II, the possibility existed that BL-expressed class II was not able to bind Ag efficiently, thus preventing T-cell stimulation. Binding assays, however, demonstrated that BL and B-LCL both bound peptide with a similar, measurable affinity. 

Bryostatin-1, a potent modulator of protein kinase C, has previously been shown to stimulate upregulation of HLA class II in colorectal cell lines and dendritic cells and to enhance Ag presentation in dendritic cells [23, 24]. To date, however, its effect on HLA class II expression and Ag presentation in lymphoid malignancies has not been evaluated. Based on this information, we sought to determine if treatment with bryostatin-1 was sufficient to enable restoration of class II-mediated Ag presentation to BL. We found that the BL cell lines Nalm-6.DR4 and Ramos.DR4 did indeed regain HLA class II Ag presentation capacity following treatment with bryostatin-1. Our assays with Ramos.DR4 cells demonstrated that restoration of HLA class II Ag presentation could partially be due to increased peptide binding by HLA class II following treatment with bryostatin-1. Additionally, protein expression analysis following bryostatin-1 treatment showed a marked increase in a 17 kDa peptidylprolyl-like protein in Nalm-6.DR4, which was expressed at very low levels in untreated Nalm-6.DR4 and expressed at high levels in 6.16.DR4.DM. This protein, when extracted and used in Ag presentation assays, enhanced class II-mediated Ag presentation in 6.16.DR4.DM cells. Thus, bryostatin-induced restoration of class II Ag presentation in BL cells was mediated by an increased expression of a peptidylprolyl-like protein. 

BL is a rapidly growing malignancy and thus requires aggressive chemotherapy to control its spread. Currently used chemotherapy regimens have achieved high cure rates in both adults and children, but treatment-associated toxicities are problematic. This issue is of particular concern for elderly and HIV-infected patients who show inferior responses and reduced tolerance of treatment-associated toxicities [40]. Treatment success has improved with the use of anti-CD20 monoclonal antibody, rituximab [41]. However, toxicities remain problematic, and the use of an immunosuppressive in HIV-infected patients is a subject of ongoing debate [42, 43]. While current treatments for BL have shown overall success, there is obvious room for improvement in the treatment of elderly and HIV-infected patients. Our future studies will continue to evaluate the role of bryostatin-1 in restoring class II-mediated Ag presentation in BL and determine the immunostimulatory role of the peptidylprolyl-like protein. A better understanding of these factors may lead to development of novel immunotherapies which could augment, lessen, or eliminate the need for toxic chemotherapies. 

## Supplementary Material

Supplemental Figure 1 shows the results of whole HSA and HSA peptide titration with Nalm-6.DR4, Ramos.DR4 and 6.16.DR4.DM cells. BL cell lines Nalm 6.DR4 and Ramos.DR4 fail to stimulate IL-2 production at concentrations 5, 10 and 20 *µ*M of whole HSA or HSA peptide, while the B-LCL 6.16.DR4.DM line shows a dose-dependent increase in levels of IL-2 production. These results suggest that BL cells possess a defect(s) in the presentation of Ag(s) to stimulate CD4+ T cells via HLA class II molecules, and this defect in Ag presentation is not dependent on antigen concentration.

## References

[1] Brady G, MacArthur GJ, Farrell PJ (2008). Epstein-Barr virus and Burkitt lymphoma. *Postgraduate Medical Journal*.

[2] Perkins AS, Friedberg JW (2008). Burkitt lymphoma in adults. *Hematology*.

[3] Biko DM, Anupindi SA, Hernandez A, Kersun L, Bellah R (2009). Childhood Burkitt lymphoma: abdominal and pelvic imaging findings. *American Journal of Roentgenology*.

[4] Yustein JT, Dang CV (2007). Biology and treatment of Burkitt’s lymphoma. *Current Opinion in Hematology*.

[5] Kawa K (2003). Diagnosis and treatment of Epstein-Barr virus-associated natural killer cell lymphoproliferative disease. *International Journal of Hematology*.

[6] Snow AL, Martinez OM (2007). Epstein-Barr virus: evasive maneuvers in the development of PTLD. *American Journal of Transplantation*.

[7] Ambinder RF (2007). Epstein-barr virus and hodgkin lymphoma. *Hematology*.

[8] Ohtsubo H, Arima N, Tei C (1999). Epstein-Barr virus involvement in T-cell malignancy: significance in adult T-cell leukemia. *Leukemia and Lymphoma*.

[9] Dang CV, O'Donnell KA, Zeller KI, Nguyen T, Osthus RC, Li F (2006). The c-Myc target gene network. *Seminars in Cancer Biology*.

[10] Bellan C, Lazzi S, Hummel M (2005). Immunoglobulin gene analysis reveals 2 distinct cells of origin for EBV-positive and EBV-negative Burkitt lymphomas. *Blood*.

[11] Dave SS, Fu K, Wright GW (2006). Molecular diagnosis of Burkitt’s lymphoma. *New England Journal of Medicine*.

[12] Gerbitz A, Mautner J, Geltinger C (1999). Deregulation of the proto-oncogene c-myc through t(8;22) translocation in Burkitt’s lymphoma. *Oncogene*.

[13] Frisan T, Levitsky V, Polack A, Masucci MG (1998). Phenotype-dependent differences in proteasome subunit composition and cleavage specificity in B cell lines. *Journal of Immunology*.

[14] Sharipo A, Imreh M, Leonchiks A, Imreh S, Masucci MG (1998). A minimal glycine-alanine repeat prevents the interaction of ubiquitinated I*κ*B*α* with the proteasome: a new mechanism for selective inhibition of proteolysis. *Nature Medicine*.

[15] Yin Y, Manoury B, Fåhraeus R (2003). Self-inhibition of synthesis and antigen presentation by Epstein-Barr virus-encoded EBNA1. *Science*.

[16] Masucci MG, Torsteinsdottir S, Colombani J, Brautbar C, Klein E, Klein G (1987). Down-regulation of class I HLA antigens and of the Epstein-Barr virus-encoded latent membrane protein in Burkitt lymphoma lines. *Proceedings of the National Academy of Sciences of the United States of America*.

[17] Gavioli R, De Campos-Lima PO, Kurilla MG, Kieff E, Klein G, Masucci MG (1992). Recognition of the Epstein-Barr virus-encoded nuclear antigens EBNA-4 and EBNA-6 by HLA-A11-restricted cytotoxic T lymphocytes: implications for down- regulation of HLA-A11 in Burkitt lymphoma. *Proceedings of the National Academy of Sciences of the United States of America*.

[18] Jilg W, Voltz R, Markert-Hahn C, Mairhofer H, Munz I, Wolf H (1991). Expression of class I major histocompatibility complex antigens in Epstein-Barr virus-carrying lymphoblastoid cell lines and Burkitt lymphoma cells. *Cancer Research*.

[19] Ressing ME, van Leeuwen D, Verreck FA (2003). Interference with T cell receptor-HLA-DR interactions by Epstein-Barr virus gp42 results in reduced T helper cell recognition. *Proceedings of the National Academy of Sciences of the United States of America*.

[20] Ressing ME, van Leeuwen D, Verreck FA (2005). Epstein-Barr virus gp42 is posttranslationally modified to produce soluble gp42 that mediates HLA class II immune evasion. *Journal of Virology*.

[21] Gao FG, Khammanivong V, Liu WJ, Leggatt GR, Frazer IH, Fernando GJP (2002). Antigen-specific CD4^+^ T-cell help is required to activate a memory CD8^+^ T cell to a fully functional tumor killer cell. *Cancer Research*.

[22] Amria S, Cameron C, Stuart R, Haque A (2008). Defects in HLA class II antigen presentation in B-cell lymphomas. *Leukemia and Lymphoma*.

[23] Do Y, Hegde VL, Nagarkatti PS, Nagarkatti M (2004). Bryostatin-1 enhances the maturation and antigen-presenting ability of murine and human dendritic cells. *Cancer Research*.

[24] Kudinov Y, Wiseman CL, Kharazi AI (2003). Phorbol myristate acetate and bryostatin 1 rescue IFN-gamma inducibility of MHC class II molecules in LS1034 colorectal carcinoma cell line. *Cancer Cell International*.

[25] Haque MA, Hawes JW, Blum JS (2001). Cysteinylation of MHC class II ligands: peptide endocytosis and reduction within APC influences T cell recognition. *Journal of Immunology*.

[26] Haque A, Hajiaghamohseni LM, Li P, Toomy K, Blum JS (2007). Invariant chain modulates HLA class II protein recycling and peptide presentation in nonprofessional antigen presenting cells. *Cellular Immunology*.

[27] Hiraiwa A, Yamanaka K, Kwok WW (1990). Structural requirements for recognition of the HLA-Dw14 class II epitope: a key HLA determinant associated with rheumatoid arthritis. *Proceedings of the National Academy of Sciences of the United States of America*.

[28] Ma C, Whiteley PE, Cameron PM (1999). Role of APC in the selection of immunodominant T cell epitopes. *Journal of Immunology*.

[29] Pathak SS, Blum JS (2000). Endocytic recycling is required for the presentation of an exogenous peptide via MHC class II molecules. *Traffic*.

[30] Haque MA, Li P, Jackson SK (2002). Absence of *γ*-interferon-inducible lysosomal thiol reductase in melanomas disrupts T cell recognition of select immunodominant epitopes. *Journal of Experimental Medicine*.

[31] Younger AR, Amria S, Jeffrey WA (2008). HLA class II antigen presentation by prostate cancer cells. *Prostate Cancer and Prostatic Diseases*.

[32] Goldstein OG, Hajiaghamohseni LM, Amria S, Sundaram K, Reddy SV, Haque A (2008). Gamma-IFN-inducible-lysosomal thiol reductase modulates acidic proteases and HLA class II antigen processing in melanoma. *Cancer Immunology, Immunotherapy*.

[33] Amria S, Hajiaghamohseni LM, Harbeson C (2008). HLA-DM negatively regulates HLA-DR4-restricted collagen pathogenic peptide presentation and T cell recognition. *European Journal of Immunology*.

[34] Hill CM, Liu A, Marshall KW (1994). Exploration of requirements for peptide binding to HLA DRB1^∗^0101 and DRB1^∗^0401. *Journal of Immunology*.

[35] Khanna R, Burrows SR, Thomson SA (1997). Class I processing-defective burkitt’s lymphoma cells are recognized efficiently by CD4^+^ EBV-specific CTLs. *Journal of Immunology*.

[36] Matloubian M, Concepcion RJ, Ahmed R (1994). CD4^+^ T cells are required to sustain CD8^+^ cytotoxic T-cell responses during chronic viral infection. *Journal of Virology*.

[37] Mi JQ, Manches O, Wang J (2006). Development of autologous cytotoxic CD4^+^ T clones in a human model of B-cell non-Hodgkin follicular lymphoma. *British Journal of Haematology*.

[38] Khalil H, Deshaies F, Bellemare-Pelletier A (2002). Class II transactivator-induced expression of HLA-DO*β* in Hela cells. *Tissue Antigens*.

[39] Staege MS, Lee SP, Frisan T (2002). MYC overexpression imposes a nonimmunogenic phenotype on Epstein-Barr virus-infected B cells. *Proceedings of the National Academy of Sciences of the United States of America*.

[40] Aldoss IT, Weisenburger DD, Fu K (2008). Adult Burkitt lymphoma: advances in diagnosis and treatment. *Oncology*.

[41] Thomas DA, Faderl S, O’Brien S (2006). Chemoimmunotherapy with hyper-CVAD plus rituximab for the treatment of adult Burkitt and Burkitt-type lymphoma or acute lymphoblastic leukemia. *Cancer*.

[42] Oriol A, Ribera JM, Bergua J (2008). High-dose chemotherapy and immunotherapy in adult Burkitt lymphoma: comparison of results in human immunodeficiency virus-infected and noninfected patients. *Cancer*.

[43] Gea-Banacloche JC (2010). Rituximab-associated infections. *Seminars in Hematology*.

