# Supplementary figures and images for: HLA Class II Defects in Burkitt Lymphoma: Bryostatin-1-Induced 17 kDa Protein Restores CD4+ T-Cell Recognition
Source: Clin Dev Immunol. 2011 Nov 28;2011:780839. doi: 10.1155/2011/780839 (PMC3227386; doi:10.1155/2011/780839)

Hossain et al. Supplemental Figure 1.

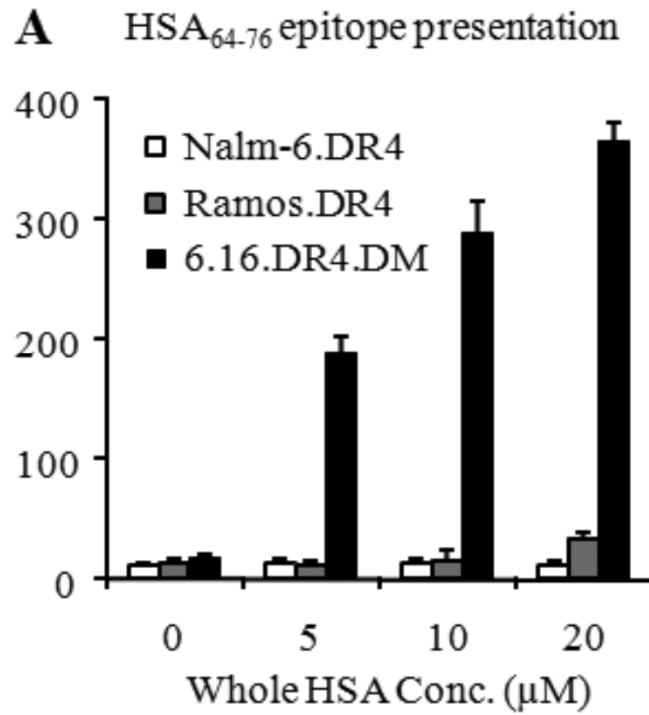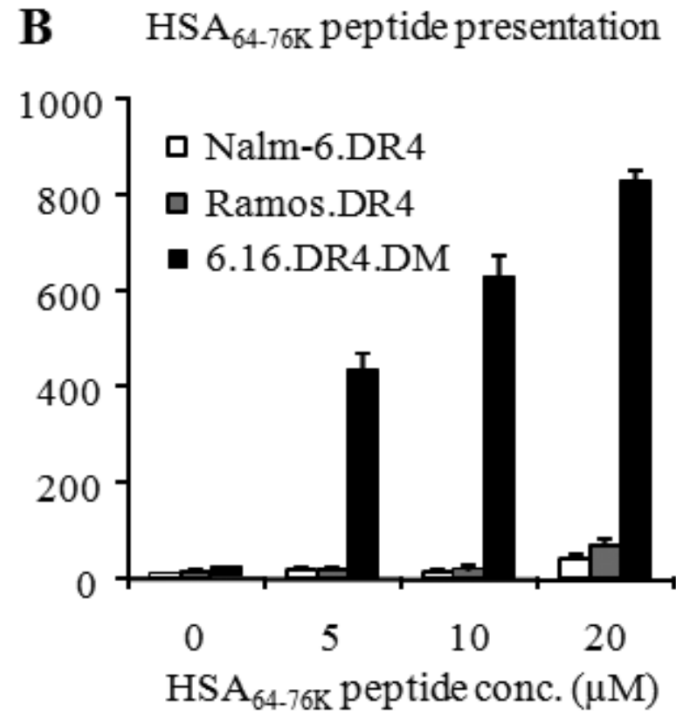

Supplement: Supplementary file 1 — Supplemental Figure 1 shows the results of whole HSA and HSA peptide titration with Nalm-6.DR4, Ramos.DR4 and 6.16.DR4.DM cells. BL cell lines Nalm 6.DR4 and Ramos.DR4 fail to stimulate IL-2 production at concentrations 5, 10 and 20 µM of whole HSA or HSA peptide, while the B-LCL 6.16.DR4.DM line shows a dose-dependent increase in levels of IL-2 production. These results suggest that BL cells possess a defect(s) in the presentation of Ag(s) to stimulate CD4+ T cells via HLA class II molecules, and this defect in Ag presentation is not dependent on antigen concentration. [file 780839.f1.pdf]
